# Conceptualisations of “good care” within informal caregiving networks for older people in rural South Africa

**DOI:** 10.1016/j.socscimed.2024.116597

**Published:** 2024-02-01

**Authors:** Michelle R. Brear, Lenore Manderson, Themby Nkovana, Guy Harling

**Affiliations:** a School of Public Health, University of the Witwatersrand (South Africa); b Monash University (Australia); c Wits Health Consortium; d University College London (United Kingdom); e Africa Health Research Institute (South Africa); f University of KwaZulu-Natal (South Africa)

**Keywords:** Caregiving, older people, ageing, care ecology, sociomateriality, ethics of care, familial care

## Abstract

Good care in social policy statements is commonly implied as familial and person-centred, provided by family members and focused on upholding the autonomy, dignity and respect of the care recipient. Policy consideration of the relational nature of caregiving, the sociomaterial determinants of good care, the practical knowledge of caregivers and responsibilities of the state, is limited. Drawing on the ethics of care theory and a care ecology framework, which conceptualises the dynamic interactions between formal and informal care “systems,” we analysed ethnographic data of the interactions of 21 caregivers and their older care recipients in South Africa to understand how they conceptualised good care. Conceptualisations of good care included: having the right, altruistic and reciprocal, motivations; providing care frequently and consistently; and demonstrating hope for a better future through practical action. Caregivers also considered restricting autonomy a feature of good care, when doing so was perceived to be in the care recipient’s best interest.

Conceptualisations of good care were influenced by but also countered policy and cultural ideals. When they subverted policy values and practices, by overriding autonomy, for instance, caregivers’ conceptualisations reflected their practical experiences of caregiving amidst gross material inadequacies, underpinned by deficiencies in the formal care system. We highlight the need for policies, interventions and theories of care that focus broadly on the care ecology and particularly on the “carescape” (formal care system). We advocate relational approaches that consider and balance the needs, desires and rights of caregivers and care recipients, and recognise caregivers’ experiential knowledge, rather than person-centred approaches that focus exclusively on the care recipient.

*[M]any older people receive care that is grossly inadequate…[focused] on keeping older people alive rather than supporting … broader objectives such as well-being and maintenance of dignity, personal choice and respect. (WHO, 2017, 8)*

Social policy statements, commissioned reports and scholarly papers frequently distinguish between good care, which is high quality, valued, dignified or person-centred, and poor care — inadequate, neglectful or abusive. Such labels are imbued with moral judgments about how care should be provided (Freeman, 2023). Policy discourse implies that care is better if “person-centred,” focused on meeting the broad needs and rights, and maximising the autonomy, of the care recipient (Pot et al., 2023). In many contexts, including in South Africa where our study was based (DSD, [South African Government Department of Social Development], 2005; DSD, 2021) and continentally (African Union, 2010, 2016) policies advocate person-centred care. “Good care” is also implied to be familial, that is, homebased and provided by unpaid relatives (African Union, 2016; DSD, 2005).

Conceptualisations of good care as based in the home and provided by kin, are assumed to be universal and always cooperative, welcomed and best able to meet the wellbeing of the person receiving care (Ang & Malhotra, 2022; Coe, 2021; Kadi et al., 2022; Kittay, 2019). These conceptualisations assume the (unlimited) capacity and willingness of family members to provide care without account of caregivers’ rights and wellbeing. However, receiving care at home is not always best for care recipients or caregivers (Kadi et al., 2022; Kittay, 2019; Laugier, 2015; Thelen, 2021). Well-intentioned caregiving can have negative outcomes for caregivers and/or care recipients, which Anna Leibling (Leibing, 2019) terms “fallacies of care”. This has, until recently, been overlooked in feminist ethical theories and anthropologies of care (Leibing, 2019; Thelen, 2021).

Relational approaches, in contrast to person-centred approaches, conceptualise caregiving as a process of giving and receiving within complex social relationships (Mitchell et al., 2020). Although the approach can perpetuate an orthodox view of care as exclusively positive (Coe, 2021; Thelen, 2021) by situating care as an “unquestioned higher value” (Leibing, 2019, 100796), relational approaches attend to caregivers rights and wellbeing and acknowledge that care recipient autonomy can result in the domination and exploitation of caregivers or others (Pols et al., 2017). A relational approach highlights the invisible, under-valued and gendered nature of caregiving, and that while the work of care largely falls to women, not all women are willing or able to provide care. The approach emphasises care provision within specific social, historic and economic contexts, and the different contributions, perspectives, needs and rights of both caregivers and care recipients (Laugier, 2015; Mitchell et al., 2020).

The attention of relational approaches to context suggests value in better knowing how the “sociomaterialities” (Lupton & Lewis, 2022) of “care ecologies” (Bowlby & McKie, 2019) influence conceptualisations of good care. Sociomateriality refers to assemblages of human and non-human actors and highlights how policies, services, conditions of everyday living, access to financial support, and so on, are entangled with and influence people’s practices (Lupton & Lewis, 2022). Care ecologies are constituted of dynamic and interactive “carescapes” (the formal or state-provided aspects of the care system) and “caringscapes” (the informal, familial aspects of the care system) (Bowlby & McKie, 2019). Each “scape” encompasses both social and material elements: networks of people involved in caregiving relationships, for example, and the physical resources available to support caregiving.

Relational approaches premise the need to value caregivers’ intimate practical knowledge of how best to give care to particular people in particular contexts. They highlight the value of caregiving research on those involved in caregiving relationships, so as to “interrogate existing ideals” (Kelly & Sebego, 2023, 16) and determine what constitutes “good” care in specific contexts (Kabelenga, 2023). Enhanced understandings of informal care provision for older people in diverse settings, where populations are rapidly ageing, has profound implications for familial care organisation, (African Union, 2016; Coe, 2021; Freeman, 2023; Kelly & Sebego, 2023; Lupton & Lewis, 2022). To this end, we explore how good care is conceptualised by rural South Africans in the caregiving networks of older people.

## Materials and methods

We draw on ethnographic data from a mixed-methods study, “Kaya”, on informal caregiving for older people with dementia in rural South Africa (Authors, 2022), which also included a survey of >1000 caregivers. The study received ethical approval from the Human Research Ethics Committee (institution and approval number withheld for review). Kaya was conducted within and used as a sampling frame people participating in a longitudinal study on ageing (Bassil et al., 2022) in the Agincourt Demographic and Health Surveillance site (hereafter Agincourt) in north-east Mpumalanga, South Africa (Kahn et al., 2012).

Under apartheid, Agincourt was part of a bantustan, a segregated and independently governed area for a specific black ethnic group, in this case Vatsonga (also known as Shangaan). Black people were forcibly removed to bantustans in the mid-20^th^ century. They needed a passbook to leave, for example, to work in a South African city, mine or commercial farm and were required to maintain a bantustan home to which they returned at least annually. Typically, younger men left to work for cash to pay bantustan taxes introduced by the apartheid government. Older people, women and children remained in bantustans to subsist on farming as best they could (Wolpe, 1972).

Life outside bantustans — in South Africa where only white people had citizenship rights — was highly regulated; women held low paying jobs in domestic and caregiving work; men worked in mines, factories and as farm labourers. (Wolpe, 1972). Public spaces and facilities were racially segregated and black people could be arrested for venturing into white areas. Within Bantustans families largely fended for themselves with limited government assistance. For example, although Older Person Grants were introduced for white people in 1928, no social grants were provided for black people until the transition to democratic governance in 1994. Education and health care were sharply segregated and discriminatory. Older people in Bantustans were cared for at home by family members; white counterparts in South Africa had access to institutional care (DSD, 2021).

Poverty and inequality have persisted since the end of apartheid in 1994, despite the extension of social development programs to black South Africans since then. These include unconditional government grants for children and older people and decentralised public health infrastructure. However, in Agincourt and other former bantustan areas, people still experience high levels of unemployment, limited education and poor health. Material deprivations continue, including limited water, sanitation and hygiene infrastructure, poor quality and crowded housing, and sparse transport infrastructure (Hoffman & Roos, 2020). High rates of HIV-related premature mortality in the 1990s and early 2000s combined with continued outmigration for work shapes the demographic profile of the area, and the availability of family members to provide care (Kahn et al., 2012). Most migrants work in unprestigious, poorly paid jobs and have limited capacity to financially support families or for socially (DSD, 2021).

As described elsewhere (Authors, 2022), we recruited 23 people (out of 25 approached) who in the Kaya survey expressed interest in participating in an in-depth study, and who identified themselves as the primary caregiver of an older person who relied on informal care and was thought to have cognitive impairment. Potential participants from seven neighbouring and interconnected villages within Agincourt were recruited through home visits and all provided written informed consent. They were sampled for maximum diversity in terms of age, gender and relationship to the care recipient. Data were collected from three male and 18 female caregivers; two other women withdrew informally from the study (i.e. by never being available). We worked intensively with five participants, with more frequent and extended home visits, reflecting their willingness and because the care recipients had higher needs, so were likely to reveal important insights about caregiving. Although our interest was on cognitive impairment and memory problems, only two of the 21 care recipients were observed or reported by their caregivers to be so affected. Others had functional impairment and health conditions which influenced their care needs, as discussed below. Hence our findings relate to caregiving for older people in general, not only those with memory problems.

Participant observation was the primary data collection method. Michelle, Author 1 a white female, and Themby, Author 3 a black female Shangaan-speaking research assistant, visited participants in their homes and sometimes accompanied them to other settings, including health facilities and funerals, from August 2022-March 2023. Interactions were predominantly in Shangaan (the participants’ first and preferred language) and were translated in the field from English to Shangaan and vice versa, by Themby. Our visits mostly involved sitting around chatting with the participant and/or their care recipient for hours, as this was their daily routine. We participated in household routines informally, following cultural norms. Occasionally we helped participants clean or fetch water, other times we watched caregivers do this, while chatting with care recipients. We sometimes exchanged food with participants and provided transport and accompanied participants to observe their experiences outside of the home.

Extensive field notes were handwritten in English during or immediately after visits, and electronically transcribed by Author 1, typically within 24 hours of the visit. We consciously focused on recording informal conversations using field notes rather than audio recording formal interviews, because we noticed participants “performing” for the audio recorder (e.g. trying to think of a right answer). At the time of writing, we had visited each household, on average, 11 times (range 3-35 visits) for 65 (range 5-430) minutes per visit.

To supplement participant observation, we conducted 17 semi-structured interviews with nine primary and six non-primary caregivers of nine different care recipients (average 39 minutes, range 7-102 minutes). Questions were developed specifically for each interview to explore specific events or aspects of caregiving. Interviewers were recorded and transcribed by Themby. Interviewers were audio recorded and transcribed by Themby. All data were compiled in Nvivo for combined analysis. Details of the participants, field notes and interviews are provided in the supplementary file as Tables 1, 2 and 3 respectively.

An abductive (switching between inductive and deductive), interpretive, ethnographic data analysis approach was used (LeCompte & Schensul, 2012). Inductively the analysis was informed by Michelle’s ethnographic field work experiences, and multiple readings of the data to identify patterns, recurring ideas and themes relevant to “good care” within and across cases. Deductively the analysis was informed by the scholarly caregiving literature and policy texts. We examined the data for latent and explicit meanings, with particular importance placed on aspects of care that participants indicated were significant to them. For example, care recipients at times become emotional when discussing an issue, repeating points, or making gestures and expressions to indicate their importance. Illustrative cases were identified and described as part of the results narrative. We discuss these findings and their implications for relational and person-centred conceptualisations of care and the sociomateriality of the “care ecology” (Bowlby & McKie, 2019).

### Findings: Good care and good caregivers

Good care was most prominently conceptualised by caregivers and care recipients as underpinned by: (1) the “right” motivations; (2) hope that their caregiving would effect positive outcomes; and (3) constant provision. A less prominent practice, (4) restricting autonomy, was considered good care for people who were perceived to be unable or unwilling to act in their own best interests.

### The “right” motivations — altruistic ideals and reciprocal realities

Having the “right” motivation was considered an important component of good care, for both caregivers and care recipients. Caregivers were constructed as ideally altruistic – concerned more about the care recipient than themselves – or at least not explicitly motivated by material reward. Somewhat paradoxically, reciprocity in the form of “giving back” to older people who had previously and/or currently provided care to others, not necessarily the caregiver, was also conceptualised as a “right” motivation. Reciprocity was the norm, in this context of material deprivation, where family interdependence in the absence of state support was established historically, in the bantustan era.

Almost all caregivers who provided care to a family member (19/21) did so without regular remuneration, even when care was a fulltime responsibility. Hayley Gumede (pseudonym), who cared for her grandmother, was the exception. She started receiving remuneration for caregiving when she married and moved out of her grandmother’s home where she had been raised. Paying Hayley acknowledged that caring for her grandmother reduced her capacity to contribute labour to her marital family, as is customary: daughters-in-law are expected to contribute caregiving and other labour to their marital families. Several older women participants lamented that their sons (and in one case grandsons) had not married and provided them with unpaid (grand)daughter-in-law caregivers. Another woman resented her daughter-in-law having “run away” to join her son and seek work in the city, rather than staying to care for her parents-in-law.

The two non-family, paid caregivers, both women, received remuneration far below the legal minimum wage for domestic workers [ZAR4400 or GBP185 monthly for 40 hours per week of work, in 2023 (South African Government Department of Employment and Labour, 2023)]. Both portrayed their caregiving as partly altruistic. For example, Sethu Gumede received a mere ZAR510 (GBP21) per month, working for Mr Peter Godi three and a half days per week. Mr Godi needed care because of severe vision impairment. Sethu was paid to cook and clean the house. Without pay, she also kept the yard tidy, planted a crop of maize and peanuts in the rainy season, helped Mr Godi access the clinic for check-ups, and collected his chronic disease medications from the local clinic. Sethu presented this work as altruistically motivated because she cared about Mr Godi, and considered what she was doing in his best interests.

Non-material rewards were also regarded as the “right” motivations for giving care. These included social rewards, such as not being gossiped about or being spoken of positively, and anticipated future rewards from God. Sethu worried that if she left the yard messy people might gossip about her, saying that she was paid but did not take good care of Mr Godi. Fanisa Mkhantswa lived next door to and cared for her father-in-law. He had minimal contact with and received virtually no care from his 13 living children. Fanisa had taken on the role of primary caregiver as “a woman of God;” she believed that her acts would result in God blessing her children. However, she also noted, “Besides, what would people say about me if I didn’t?” — caregiving for her father-in-law was a way of maintaining her social status in the eyes of community members, as well as maintaining her “self-understanding as a morally and socially worthy person” (Kittay, 2019, 48).

Participants also spoke of or alluded to reciprocity or “giving back” to parents and grandparents who had provided care in the past. Six of the primary caregivers were grandchildren (one grandson, five granddaughters) and five were children (two sons, one daughter and two daughters-in-law) of the care recipients. In the study area, many grandchildren had been raised by their grandparents either because of parental death or because their parents had migrated to cities to work. For example, 23-year-old Enelo Ngubeni had lived with her mother in an urban centre approximately seven hours drive and a ZAR380 public transport fare away from Agincourt. Enelo told us that when her grandparents’ health deteriorated, they asked her to come back to live with and care for them, because she was their favourite grandchild. When we met Enelo she had two young children of her own, cared for and financially provided for in her grandparents’ home (a third was born during the study). Enelo did not mention her grandparents’ reciprocal financial care, but her grandmother complained several times that she and her husband were financially stressed because they covered the costs of providing food for all household members, including grandchildren (Enelo and two of her brothers) and great-grandchildren (Enelo’s three daughters and her sister’s two children). The grandmother once suggested it would be cheaper to hire a caregiver than care financially for these eight household members. She also thought having a paid caregiver might be better because family care, provided for love not money, was outside of her control.

Enelo was one of many caregivers who received reciprocal support from care recipients through childcare and/or “financial care” (Button & Ncapai, 2019, 560). Yet caregivers did not acknowledge this reciprocity; they masked the financial care they received from care recipients, subtly implying that their caregiving was not motivated by financial need, and perpetuating altruism as the ideal motivation. However, because of high levels of unemployment and poverty, financial and material support (e.g. shelter) from care recipients was essential: most grandchild and child caregivers had no steady income of their own, or relied on childcare grants of ZAR500 per child per month or ZAR350 per month COVID relief grants as their only regular income. Only one caregiver had steady employment. She received <ZAR1000 per month working full time as a child caregiver. Without financial independence, younger caregivers relied on their care recipients — parents and grandparents — for food and shelter.

Violet Sibuyi, a younger wife who cared for her husband, relied on him financially because she was not yet eligible to receive an Older Persons Grant. Two older wife caregivers received an Older Person Grant but still depended on their husbands or other male relatives for housing, because, consistent with Agincourt’s chiefly system of governance and land allocation, the homesteads in which they lived were perceived to be the property of their husbands. Doris Nkuna, who cared for her husband, told us that her brother-in-law had threatened to disinherit her when she sent her husband to the clinic with their daughter instead of accompanying him herself. Doris told us she wished she had never married and wished she could leave her husband. However, she had nowhere to go.

Only one participant, Isaac Silaule, spontaneously mentioned relying financially on the person for whom he cared, his mother. Isaac was 37-years-old and earnt sporadic income doing “piece jobs” in the construction industry. His mother paid for food; he used his own income to buy personal items such as clothing and gifts for his mother such as a television.

Several grandchildren suddenly moved and ended their caregiving when they found employment. Although it was often unskilled and poorly paid, employment reduced their dependence on their grandparents. Many of these grandchildren had previously reported altruistic or reciprocal motivations for caregiving, and commitment to continue caring for their grandparents indefinitely. For example, Hetisani Dlamini, a granddaughter caregiver, responded to our question about why she gave care, with “How can I stop?” She explained that, in the year before we met, her grandfather and she had together experienced the death of her mother and grandmother. A month later, Hetisani moved to Gauteng to start work in a supermarket. She was one of four granddaughters (including Enelo, discussed above) and two grandsons (one primary, the other a secondary caregiver), who stopped caring for their grandparent/s or limited their caregiving, either permanently or temporarily, when they found paid work.

### Hope - prolonging and optimising the quality of life

Maintaining hope that the care recipient’s condition would improve, and that the future would bring better days, was also an important feature of good caregiving. This was expressed in a range of practices enacted to prolong or enhance the older people’s quality of life, most prominently food provision, keeping care recipients clean and nicely dressed, and actively seeking treatment for them.

Food provision, including buying and preparing food and sometimes assisting a care recipient to eat, was a central act of good care and an expression of hope for a longer, better quality life. It was also a way for caregivers to express hope of recovery from and/or to prevent (worsening) illness. Caregivers went to considerable effort, for example cooking outdoors over fires, to ensure food was ready when care recipients needed to eat, such as before taking chronic disease medications. Several caregivers also restricted the food choices of care recipients with diabetes, either regularly or irregularly. For example, they limited the amount of salt and oil they put in the care recipients’ cooked food and restricted their access to soft drinks and sugary foods, even though the diabetic care recipient loved these *mahinyahinya* (Shangaan: “nice food” or treats).

In other cases, good care included providing *mahinyahinya* such as ice cream, cornflakes and milk, rolled oats, sweets, biscuits, fresh fruit, hot chips and soft drink. Caregivers spoke of giving the healthier of these foods (e.g. fruit, oats) as well as staples such as maize meal porridge and indigenous spinach as therapeutics — foods they believed would heal the body, promote health and/or provide energy and strength. Real *mahinyahinya* was a treat, typically purchased with scarce resources, enjoyed for the taste and shared with others. It was provided to care recipients in the hope of making them feel good, albeit only temporarily.

Providing food as an act of hope for prolonged life was most poignantly demonstrated when a gravely ill care recipient, Xiluva Shabangu, refused food regularly for several weeks before she died. When she could, Xiluva’s sister and primary caregiver Vangama would stash and later give Xiluva treats like ginger biscuits, and mangoes she harvested from her trees. She knew Xiluva would eat these foods, whereas she often refused the healthy but bland staple meals of hard maize porridge and greens. One day we visited around 11am, to hear from Vangama and Xiluva’s daughter and secondary caregiver Kayise that she had not eaten all morning. We sat with Vangama and Kayise, watching Xiluva shifting on her bed, unable to find comfort for what seemed like hours but was perhaps 20 minutes. Several of us became teary and we barely spoke. Vangama eventually broke our hopeless trance by shaking her sister roughly. She told Xiluva to wake up, that her friends from the university had come to visit, and had bought her some bananas to eat. Xiluva roused, sat up and ate a banana. Our moods lifted because, as Kayise commented, “at least when she is eating, we can hope.”

Hope for the care recipient’s wellbeing was also expressed in keeping them clean and well-dressed. This involved far more than helping with dressing. It also meant supplying clothes and keeping clothes and bodies clean. This was a challenge in a community where water was rarely piped through taps more than once per week; for some households no water was piped through taps for the entire study period. Most participants reported, or were observed, struggling to secure enough water to keep clothing, bedding and the caregiving environment clean. Many reported using limited financial resources to pay exorbitant prices for water deliveries, walking long distances to collect water in 200 litre drums which they rolled to and from the water source, waiting for hours at the roadside for government water tanker deliveries, and/or harvesting water from roofs into buckets and other receptacles during the raining season. While water procurement was part of a broader domestic economy, it was conceptualised as playing a specific role in caregiving, especially for people who couldn’t bath or fetch water themselves — it conferred dignity and happiness on the care recipient, because bathing felt good and made them “fit” to interact with other people.

Actively seeking therapy that could prolong and improve quality of life was integral to caregivers and care recipients maintaining hope. It centred around attempting to access biomedical treatments that might improve the care recipient’s and/or caregiver’s health or prolong their life, including medications to control chronic diseases and relieve pain. Although many medicines were available free of charge to older people from public health clinics, access often involved considerable effort and expense because of the need to travel (typically 4-8 kilometres) to health clinics and wait for hours in long queues. Four caregivers and 10 care recipients used anti-hypertensive medication; they also accessed health care for gastrointestinal conditions (4), kidney disease (1), diabetes (2), HIV (4), tuberculosis (2), hot flushes (1), vision problems (12) and pain (11) possibly caused by arthritis. Several older caregivers sought biomedical therapy for themselves as an act of caregiving, because without treatment their ability to provide care would decline.

### Consistency - never getting tired and always being there

“Other women would have run away and left him,” Violet Sibuyi told us when we first chatted about her role as primary caregiver for her husband. Mr Sibuyi’s kidneys had failed some ten years before we met Violet. He died several months later. “His family thanked me for sticking by his side until the end,” Violet told us when we first saw her after the funeral. She emphasised, and seemed to find comfort in knowing, that she was at her husband’s side when he took his final breath, evidence that she had succeeded in providing him with “good care” until the end. She related her tenacity to continue to provide care and know what to expect to the teachings of widows at the Zion Church of Christ. The church ran weekday lessons to prepare women congregants to give care to ailing and ageing husbands.

Other participants referred to “never getting tired” as a positive feature of their own or others’ caregiving. Vangama Ndubane — aged in her 80s or 90s, depending on the source -- told us with tiredness written all over her face that she would never get tired of bathing her younger sister Xiluva, who was in her 80s. Vangama was at Xiluva’s bedside when she passed away, six months after we met the sisters and eight months after Vangama became Xiluva’s primary caregiver. Vangama’s testimony suggests “never getting tired” means never leaving the caregiving role and continuing the physical labour of care, despite physical and emotional exhaustion.

Both Violet and Vangama told us not to worry about phoning before coming to visit, because we would always find them at home, by their care recipients’ sides. Like most other caregivers, they did go out sometimes, but the notion that good caregivers devoted all their time to care recipients was common. Always being there was an especially important characteristic of good care for one care recipient (seemingly with undiagnosed dementia) who wandered and got lost, and/or a few who needed assistance with daily living activities such as toileting and eating. For example, Violet’s husband Xongela could only walk with the assistance of a stick. To reach the homestead pit latrine, some 25 metres from the back door of the house, Xongela needed to use his stick and lean on someone, as he navigated three steps and the sloping, uneven terrain. Violet’s adult sons helped Xongela, their father, with toileting and other tasks, when she needed to go out. Other caregivers, because of their caregiving responsibilities, reported or were observed not doing things they needed or wanted to do — accessing health services, visiting relatives at Christmas time, finding employment, socialising, having intimate relationships, doing community-based volunteer work, resting, or attending church.

### Overriding autonomy - locking in, coercing and restricting care recipients

Another important feature of good care was the willingness of the caregiver to override the care recipient’s autonomy or expressed choices, when this was perceived to be in their best interests. We observed and were told of numerous practices that involved overriding autonomy – locking care recipients in; coercing them to bathe, eat or access medical care, sometimes by lying or making threats; and restricting their consumption and spending choices.

Worried *that her* sister Rhandzu would get lost or harmed, Xisthembiso Nkuna locked the gate with a chain and padlock to prevent Rhandzu from getting out and walking off. Rhandzu was the only care recipient in our study who had behavioural symptoms consistent with moderate-severe dementia, and she wandered off twice in the study period, once before Xisthembiso started locking the gate. On the second occasion, Rhandzu woke early and “stole” the keys from her sleeping sister’s bedside and escaped. While walking in the neighbourhood, she fell over and grazed her leg, arm and face. Rhandzu arrived at her brother’s house, approximately 30 minutes’ walk away, four hours after Xisthembiso had woken to find her missing and had started searching for her.

Several caregivers threatened or lied to care recipients to get them to eat or bathe. Coercion was always constructed as a component of good care, in the care recipients’ best interests. When Xiluva (discussed above) refused to eat, her caregivers would pretend to call the hospital or police and arrange to have Xiluva taken away if she refused the food they had prepared. One day Xisthembiso, trying to encourage Rhandzu to eat, told her that we really wanted her to eat and would be happy if she finished her food. Rhandzu replied tartly that she could still understand Xitsonga/Shangaan, and she knew we had not said that. She ate the meal spoon-fed to her anyway. Caregivers also encouraged care recipients to bathe by stretching the truth. For example, one reported that she would encourage her care recipient to bathe by telling her that we were coming to visit, and so she must get dressed and look nice.

Caregivers also restricted or overrode care recipients’ choices and framed doing so as an act of good care which protected them from harm. Doris Nkuna’s daughter ignored her request to go home because she was tired and felt that the nurses who kept her waiting for hours, were disrespecting her. She insisted Doris wait at hospital to be prescribed anti-hypertensive medicines, which were effective in reducing the swelling and pussy sores Doris had on her feet, diagnosed by the doctor as complications of untreated hypertension. Younger caregivers often withdrew and shopped with an older person’s grant money, under informal agreement. We never heard of any caregiver having a legal entitlement such as power of attorney, to make financial decisions for a care recipient. Yet, caregivers restricted choices about how care recipients spent their Older Person Grant money. Violet Sibuyi reported approvingly that her neighbour’s daughter would give her mother only ZAR500 of her Older Person Grant, because the neighbour would spend all the money on alcohol. The daughter used the grant money instead to buy food and other items that she considered essential for her mother’s wellbeing.

One paid caregiver, Fatima Chauke, reported that her care recipient’s, Katekani Mabaso’s, son managed her money. Restricting Katekani’s access to her grant money was again to prevent her spending it on alcohol, which she would drink in large quantities when available. Katekani was disinhibited when she drank. She would call Fatima names and purposely defecate in the house. Katekani complained about her son “stealing” her money. She was especially irritated that he refused to use her social grant money to buy her cask wine.

About six months after we met Katekani, some 10 months after Fatima became her live-in primary caregiver, Katekani decided to give up drinking and using snuff. She stopped being rude to Fatima and complaining about her son stealing her grant money too. Fatima thought (and convinced us) that this was because she was providing Katekani with good care. Relatives had drawn this to Katekani’s attention when visiting over Christmas, commenting that Katekani was clean and well dressed, had gained weight and looked well fed. We had also noticed these changes, and Katekani herself often commented on what good care Fatima provided — mentioning acts such as cooking delicious food, heating bath water and assisting her to bathe, and staying with her day and night. She did not want to lose her.

## Discussion

The study involved a small number of participants and ethnographic analysis (LeCompte & Schensul, 2012), undertaken predominantly by the first author. Her close relationships with participants, developed over time, enabled us to interrogate good care at multiple points in time, as the caregiving environments, care recipients and caregivers changed (e.g. in response to events and moods), bringing into focus different aspects of caregiving and elucidating various reasons why caregiving was practiced as it was. The results offer a complex and dynamic picture of how caregivers and care recipients conceptualised “good” care. While these conceptualisations sometimes concur with dominant discourses, they often extend and at times contradict practices upheld as characteristic of good care in hegemonic discourses. We now turn to discussing the implications of our findings for policy and practice, with reference to care ecologies and “fallacies of care.”

### Keeping older people alive amidst gross inadequacies

The World Health Organization (WHO, 2017), quoted as the epigraph, labels caregiving focused merely on keeping people alive as “grossly inadequate.” It juxtaposes such caregiving with person-centred care, which aims to uphold care recipient’s dignity, choice and respect. Our results indicate that in precarious care ecologies characterised by material (e.g. food and water) and social inadequacies, keeping older people alive is a prerequisite for and in itself represents caregivers’ best attempts to enhance older care recipients’ wellbeing, and maintain their dignity, choices and respect.

Older people’s and their caregivers’ choices (autonomy and agency) were constrained by sociomaterial inadequacies in carescapes and caringscapes (Bowlby & McKie, 2019). These inadequacies often meant that caregivers’ and care recipients’ only available choice was between two undesirable options: eating food that was difficult to swallow or not eating (Xiluva); waiting and being disrespected by health workers or going home untreated (Doris); locking someone in or leaving them exposed to potential dangers (Rhandzu). Doris’ desire not to wait for treatment that she urgently needed was influenced by a formal health system that denied her dignity and was physically exhausting. She did not have a “free” choice. She wanted treatment for pain and swelling affecting her feet. She ultimately felt she benefited from the medicines prescribed by the doctor, but she would never have received these had her daughter respected her “choice” to go home. Doris had lost hope that she would receive help from the nurses who kept her waiting. Hope has been identified as an important aspect of good care for younger adults affected by HIV (Stadler, 2021). Caregivers in our study expressed hope of prolonging and improving the quality of life, through practices that kept older people alive and in relative comfort; this is somewhat surprising given the hegemonic constructions of older people as dependent and waiting to die.

Other autonomy-restricting practices also reflected caregivers’ best attempts to provide good care by optimising care recipient wellbeing despite poverty. Caregivers controlled money so that older people did not spend it on alcohol, both to enhance dignity and ensure that sufficient money was available for essentials such as food. They restricted food choices of diabetic patients. In each case, restricting autonomy was enacted because it was believed to be in the care recipient’s best interest.

The concept of fallacies of care is an is analytical approach for problematising well-intentioned caregiving practices (Leibing, 2019). It implies that what caregivers believe to be in the best interests of care recipients, is mistaken, if it contradicts hegemonic constructions of good care. Conversely, the beliefs of caregivers in our study, reflected their intimate practical knowledge of care recipients and the sociomaterial context of the ecology in which they provided care. “Beliefs” about what constituted “good care” and practices to enact good care, were tailored to the individual care recipient and their context. Xisthembiso locked the gate based on past experience of her sister wandering, getting lost and being harmed, albeit only minor cuts and scratches. Vangama stashed and bought nice foods when she could because she knew her sister would eat these willingly. She coerced Xiluva to eat only when desirable food were unavailable. Using her practical knowledge Vangama was able to “attend to difference” in ways that respected autonomy (Driessen & Ibáñez Martín, 2020) study. The care provided by Vangama, Xisthembiso and other caregivers provided, drawing on their experiential caregiving knowledge, was not only optimal given the structural constraints they faced but was based on logical reasoning, informed by intimate knowledge of the context and care recipient.

Caregivers’ tasks were exacerbated, and the quality of care they provided undermined, by material inadequacies, which they were powerless to change. These included food and water insecurity, lack of transport, and built home environments not conducive to the changing abilities of care recipients. For example, care recipients needed assistance to walk down stairs and across rugged terrain to access pit latrines. They may have been able to accomplish such tasks independently (and might reasonably have chosen to do this and felt that it enhanced their dignity), if their caringscape included an indoor flushing toilet and reliable water supply. Food, although often sufficient, was typically basic and not what care recipients might have chosen, if a choice was possible. Changing the types of food available might have been more effective than threatening them to eat, less work for caregivers, and more attentive to care recipients’ identities (Driessen & Ibáñez Martín, 2020). However, preferred food were often unavailable for financial reasons.

When fresh fruits (or other “treat” foods) were available, feeding went well beyond keeping older people alive. Growing, buying, preparing and/or giving *mahinyahinya* was, for caregivers in our study, as Brijnath (2011) found in India, a way of maintaining and enhancing relationships. It also respected autonomy by attending to difference, by providing or knowing individual choices as in (Driessen & Ibáñez Martín, 2020). However, the third element of attending to difference, “catering to identities” (Driessen & Ibáñez Martín, 2020) was often not possible because of material constraints. Providing nice food or other pleasurable experiences such as baths, were also ways for caregivers to express affection and respect for the older people they cared for, as well as hope for a better future — a healthier, more enjoyable, longer life (Stadler, 2021).

The autonomy, agency and dignity of care recipients is profoundly influenced, and sometimes undermined, by material inadequacies such as lack of food water and barriers to accessing health services. These arguably should be provided within the carescape (formal care system), by the governments that have guaranteed their citizens basic rights. This state duty to care for older people and achieve broader objectives such as autonomy and dignity, is downplayed in euphemistic references to the government’s role being to strengthen or support families in South African (DSD, 2005) and continental (African Union, 2016) policies that guide the arrangement of older people’s care. Policies that advocate person-centred familial care absolve governments of responsibility and place the onus on individual caregivers and families to uphold care recipients’ dignity and choices. They imply that inadequacies in care are the fault of the caregiver/s, and neglect structural inadequacies of care ecologies that individual caregivers are powerless to change.

Simultaneously, caregivers’ practical knowledge, when it differs from policy and other hegemonic discourses, is relegated to the realm of “mistaken belief” by concepts such as “fallacies of care” (Leibing, 2019). When applied to informal caregivers, this approach assume deficits in their knowledge and practice. Rather than a fallacy — mistaken belief based on failed reasoning — caregivers’ beliefs were rational responses to precarity and structural violence over which they had limited power. Caregivers’ choices about taking on care were highly constrained because of financial dependence, notwithstanding their willingness to make sacrifices for their care recipients. To provide care they often had no option but to rely on their care recipient financially. Even if they had not been occupied full time with caregiving, getting a job and gaining financial independence was impossible for most, given extensive unemployment and exploitative work conditions.

### Family interdependency and “rules” governing who cares

The interdependencies of family members in this structurally marginalised community fit with relational conceptualisations of care. Caregivers and care recipients gave to and took from each other, out of necessity as much as choice. The process of caregiving created belonging and extended relationships of care recipients and caregivers (Thelen, 2021). These relations provide a backdrop to the valorisation of familial care. Although often presented as motivated exclusively by altruism — and in our study was motivated partly by loving and caring about the care recipient — providing care to older relatives was also a way for younger family members to develop positive social identities (Kittay, 2019) and/or reinforce their belonging to family (Thelen & Coe, 2019). This legitimised their claim to financial and other types of material care needed to maintain their own lives and caregiving capacity. However, the Older Person’s Grant was less than half of the minimum wage. The minimal funding (ZAR510 monthly) for caregivers added to the old age grant (South African Government, 2023) — a fraction of the minimum wage — reinforces the notion that caregiving should be altruistically motivated and performed without or for only minimal pay. Older people whose sole source of income is the Older Person Grant, and who often need care for more than 40 hours per week, cannot afford to pay a caregiver the legal minimum wage. Material inadequacies perpetuate the financial exploitation of caregivers who are predominantly unpaid female relatives who rely on older care recipients to fulfil their own needs.

The valorisation of family-based and altruistically or reciprocally motivated care, provided predominantly by women, was prominent in our conversations. Although no participant was familiar with the South African Policy for Older Persons (DSD, 2005), or related policies such as the Revised White Paper on Families (DSD, 2021) their doctrines of gendered, familial obligation to care was reinforced in caregivers’ everyday social interactions in church, community and home. It was also reinforced by everyday circumstances – most care recipients had no choice but to rely on unpaid family members as caregivers. Those without family members to provide care, relied on women paid a fraction of the minimum wage. Both men and women, caregivers and care recipients expected women in the family to provide care without pay. Daughters, granddaughters and daughters-in-law were especially expected to care, and if they were not available then care work responsibility fell to wives, sons or grandsons. Even so, Fatima and Sethu’s cases show that non-family caregivers could be accepted and provide good care. This finding is important in the African context, where policy rhetoric implies African families are uniquely positioned to care for elders (Freeman, 2023), that cultural values are counterposed to anything but familial care, and that gaps in older person’s care are due to the erosion of cultural values (African Union, 2010, 2016).

Amongst our participants, unwritten “asking rules” (normative expectations about when to ask for care) within the caringscape (informal care system) (Bowlby & McKie, 2019) were highly gendered. As Freeman (2023) notes: good caregivers were assumed to be female relatives, with an obligation and a disposition to care selflessly. However, norms about asking were also strongly influenced by individuals’ capability to contribute materially (especially financially) to the family. Four young female caregivers stepped out of their caregiving roles when they found paid work. Care recipients generally accepted this because they found providing financial care with only the social grant for income a burden. Grandchild caregivers were often asked or chosen for the caregiver role because their parents had moved away to work, which enabled parents to contribute materially to the family; unemployed youth could not. This meant that the least powerful family members, without income and with little chance of finding a job, were “asked” to take on caregiving in exchange for subsistence. In this context, deciding to take on a caregiving role was no more a free choice for youth caregivers than leaving the hospital untreated was for older care recipients.

## Conclusion

Within a care ecology, carescapes and caringscapes interact dynamically, but also each have internal dynamics that influence who provides care and how. Family caregivers’ and care recipients’ beliefs and practices are shaped by experiential knowledge of what is realistic and possible, and are reasoned and logical responses to material deprivations and precarity. In contexts of sociomaterial deprivation such as the former bantustans in rural South Africa, we see a need for policies and interventions that focus broadly on developing care ecologies that are materially conducive to the wellbeing of caregivers and older care recipients, rather than those that advocate person-centred care with a narrow focus on the care recipient. In policies and reports that advocate familial, person-centred caregiving, the onus for attaining and transcending older care recipients’ rights is placed on informal caregivers within the caringscape. A shift in focus to the care ecology, based on an understanding that material deprivations that can only be addressed with changes in the carescape, is needed to optimise the wellbeing, and ensure the rights, of both caregivers and care recipients.

Caregivers are often female family members, whose “willingness” to care is heavily influenced by gendered discourses as well as poverty. Changes in carescapes, that reduce precarity of caringscapes (e.g. ensure food and water security), are essential, if care is to achieve the broad objectives outlined by the World Health Organization. However, alternatives to familial care provided in the carescape should also be identified and supported. Our results highlight that, regardless of policy rhetoric that situates African family values as unique, good care can be provided by non-family members in African care settings. But financial resources from the state are needed to pay for and provide the material bases to optimise such care.

In our study context, “good” caregiving for older persons was conceptualised as part of a reciprocal exchange that enabled younger people, including non-family paid caregivers, to access essentials such as food and shelter, and/or positive social identities associated with providing good care. Caregiving was relational and involved caregivers and care recipients giving and taking what they needed, in a context of constrained resources. Acknowledging the relational nature of caregiving, and how people’s relationships are influenced both by other people and by material circumstances, could improve social policies guiding care of older persons. Legitimising the knowledge of, and learning from caregivers with firsthand experience, rather than assuming deficiencies in their knowledge and practice if it diverges from hegemonic, abstract conceptualisations of “good care”, is essential. Situating responsibility for older people’s care with informal caregivers, without extending them some power determine what constitutes good care, counters achieving good care for older people.

## Supplementary Material

Supplementary Materials

## Highlights

- Caregivers’ conceptualisations of good care were logical, based on practical knowledge.
- Caregivers’ situated practical knowledge of context and care recipient informs good care practice.
- Policies and programs focused on reducing material deprivations can optimise care.
- Good care can be provided by non-familial caregivers in this African setting.
