# Supplementary material for: Conceptualisations of “good care” within informal caregiving networks for older people in rural South Africa
Source: Soc Sci Med. Author manuscript; Available in PMC 2024 Jun 17. (PMC7616109; doi:10.1016/j.socscimed.2024.116597)
Supplement: Supplementary Materials [file EMS196557-supplement-Supplementary_Materials.docx]

**Supplementary file: Conceptualisations of “good care” within caregiving networks for older people in rural South Africa**

**Table 1: Participant (primary caregiver) and care recipient characteristics**

|  | **Primary Caregiver** |  |  |  | **Care recipient** |  |  |  |  |
| --- | --- | --- | --- | --- | --- | --- | --- | --- | --- |
| *Reference* | *Pseudonym* | *Age* | *Gender* | *Relationship to care recipient* | *Pseudonym* | *Age* | *Gender* | *N visits* | *Visits duration (mins)* |
| 01 | Khensani Mkhantswa | 28 | Female | Granddaughter | Fay Ubisi | 92 | Female | 3 | 210 |
| 02 | Hayley Gumede | 26 | Female | Granddaughter | Gladys Gumede | 80 | Female | 12 | 285 |
| 03* | Masana Mnisi | 24 | Female | Granddaughter | Hlekani Mnisi | 102 | Female | 7 | 760 |
| 04 | Isaac Silaule | 37 | Male | Son | WelaniSilaule | 67 | Female | 6 | 130 |
| 05 | Lulama Mhlongo | 57 | Female | Unrelated, paid | Peter Godi | 65 | Male | 7 | 490 |
| 06 | LulekaManyike | 29 | Male | Grandson | Rose Mhlanga | 65 | Female | 6 | 425 |
| 07 | Saseka Mathe | 28 | Female | Granddaughter | Namiah Mathe | 92 | Female | 4 | 105 |
| 08 | Tinyiko Nkuna | 44 | Female | Daughter | Job Nkuna | 94 | Male | 4 | 220 |
| 09 | Vukona Ngubeni | 37 | Female | Niece | Harry Mathonsi | 65 | Male | 7 | 160 |
| 10* | Doris Nkuna | 81 | Female | Wife | Samson Nkuna | 82 | Male | 19 | 2380 |
| 11 | Violet Sibuyi | 56 | Female | Wife | Hilton Sibuyi | 72 | Male | 5 | 290 |
| 12* | VangamaNdubane | 90 | Female | Sister | Xiluva Shabangu | 84 | Female | 34 | 3630 |
| 13* | Dorothy Mkhantswa | 72 | Female | Wife | Adam Ubisi | 84 | Male | 14 | 1575 |
| 14 | Hetisani Dlamini | 21 | Female | Granddaughter | Reason Mnisi | 82 | Male | 10 | 390 |
| 15 | Vutivi Mdaka | 61 | Female | Cousin-in-law | Nhlelo Godi | 83 | Male | 7 | 245 |
| 16 | FanisaMkhantswa | 58 | Female | Daughter-in-law | Jimmy Mkhantswa | 85 | Male | 7 | 305 |
| 17 | Themba Malamule | 46 | Male | Son | SingilaMalamule | 80 | Female | 7 | 270 |
| 18* | Xisthembiso Nkuna | 69 | Female | Sister | Rhandzu Mkhantswa | 72 | Female | 13 | 1140 |
| 19* | Fatima Chauke | 47 | Female | Unrelated, paid | Katekani Mabaso | 82 | Female | 17 | 625 |
| 20 | Enelo Ngubeni | 23 | Female | Granddaughter | Ponisa Ngubeni | 83 | Female | 14 | 670 |
| 21 | Nonisa Nkuna | 59 | Female | Daughter-in-law | Rhulani Nkuna | 94 | Male | 10 | 405 |
| * Participant followed intensively | | | | | | | | | |

**Table 2: Characteristics of fieldnotes dataset**

| **Visit number** | **Date** | **Participant reference** | **Visit duration** |
| --- | --- | --- | --- |
| 1 | 28/7/2022 | 3 | 110 |
| 2 | 10/8/2022 | 14 | 60 |
| 3 | 11/8/2022 | 19 | 75 |
| 4 | 12/8/2022 | 10 | 75 |
| 5 | 12/8/2022 | 20 | 10 |
| 6 | 12/8/2022 | 19 | 5 |
| 7 | 19/8/2022 | 20 | 75 |
| 8 | 19/8/2022 | 13 | 70 |
| 9 | 25/8/2022 | 3 | 20 |
| 10 | 25/8/2022 | 10 | 15 |
| 11 | 25/8/2022 | 19 | 20 |
| 12 | 26/8/2022 | 2 | 45 |
| 13 | 26/8/2022 | 12 | 90 |
| 14 | 27/8/2022 | 13 | 15 |
| 15 | 29/8/2022 | 10 | 165 |
| 16 | 29/8/2022 | 20 | 45 |
| 17 | 30/8/2022 | 17 | 105 |
| 18 | 30/8/2022 | 2 | 30 |
| 19 | 30/8/2022 | 14 | 5 |
| 20 | 01/9/2022 | 20 | 5 |
| 21 | 01/9/2022 | 19 | 100 |
| 22 | 02/9/2022 | 19 | 50 |
| 23 | 02/9/2022 | 12 | 5 |
| 24 | 05/9/2022 | 20 | 85 |
| 25 | 06/9/2022 | 14 | 90 |
| 26 | 06/9/2022 | 12 | 105 |
| 27 | 08/9/2022 | 13 | 50 |
| 28 | 28/9/2022 | 10 | 35 |
| 29 | 28/9/2022 | 13 | 90 |
| 30 | 28/9/2022 | 20 | 10 |
| 31 | 28/9/2022 | 3 | 100 |
| 32 | 28/9/2022 | 19 | 5 |
| 33 | 29/9/2022 | 12 | 125 |
| 34 | 29/9/2022 | 6 | 35 |
| 35 | 09/9/2022 | 2 | 10 |
| 36 | 09/9/2022 | 12 | 15 |
| 37 | 05/10/2022 | 6 | 35 |
| 38 | 05/10/2022 | 2 | 20 |
| 39 | 06/10/2022 | 19 | 30 |
| 40 | 06/9/2022 | 17 | 5 |
| 41 | 06/10/2022 | 18 | 80 |
| 42 | 06/10/2022 | 2 | 20 |
| 43 | 06/10/2022 | 13 | 30 |
| 44 | 07/10/2022 | 20 | 5 |
| 45 | 07/10/2022 | 12 | 30 |
| 46 | 07/10/2022 | 16 | 55 |
| 47 | 11/10/2022 | 9 | 55 |
| 48 | 11/10/2022 | 4 | 60 |
| 49 | 11/10/2022 | 14 | 55 |
| 50 | 12/10/2022 | 21 | 105 |
| 51 | 12/10/2022 | 10 | 75 |
| 52 | 12/10/2022 | 19 | 10 |
| 53 | 13/10/2022 | 6 | 90 |
| 54 | 13/10/2022 | 7 | 30 |
| 55 | 19/10/2022 | 1 | 65 |
| 56 | 19/10/2022 | 20 | 55 |
| 57 | 20/10/2022 | 10 | 210 |
| 58 | 20/10/2022 | 18 | 5 |
| 59 | 20/10/2022 | 2 | 10 |
| 60 | 20/10/2022 | 15 | 15 |
| 61 | 27/10/2022 | 12 | 20 |
| 62 | 27/10/2022 | 8 | 45 |
| 63 | 27/10/2022 | 3 | 135 |
| 64 | 25/10/2022 | 16 | 20 |
| 65 | 25/10/2022 | 21 | 25 |
| 66 | 25/10/2022 | 9 | 50 |
| 67 | 25/10/2022 | 15 | 5 |
| 68 | 28/10/2022 | 13 | 35 |
| 69 | 02/11/2022 | 18 | 25 |
| 70 | 02/11/2022 | 4 | 5 |
| 71 | 02/11/2022 | 19 | 45 |
| 72 | 03/11/2022 | 11 | 5 |
| 73 | 03/11/2022 | 10 | 100 |
| 74 | 04/11/2022 | 11 | 80 |
| 75 | 04/11/2022 | 6 | 100 |
| 76 | 07/11/2022 | 7 | 45 |
| 77 | 08/11/2022 | 5 | 55 |
| 78 | 08/11/2022 | 17 | 30 |
| 79 | 08/11/2022 | 18 | 110 |
| 80 | 08/11/2022 | 7 | 5 |
| 81 | 08/11/2022 | 4 | 1 |
| 82 | 11/11/2022 | 13 | 540 |
| 83 | 11/11/2022 | 13 | 105 |
| 84 | 15/11/2022 | 4 | 10 |
| 85 | 15/11/2022 | 2 | 20 |
| 86 | 17/11/2022 | 3 | 230 |
| 87 | 18/11/2022 | 20 | 70 |
| 88 | 23/11/2022 | 12 | 240 |
| 89 | 02/12/2022 | 11 | 175 |
| 90 | 06/12/2022 | 16 | 15 |
| 91 | 06/12/2022 | 19 | 35 |
| 92 | 06/12/2022 | 10 | 80 |
| 93 | 06/12/2022 | 1 | 20 |
| 94 | 06/12/2022 | 21 | 5 |
| 95 | 07/12/2022 | 9 | 5 |
| 96 | 07/12/2022 | 17 | 15 |
| 97 | 07/12/2022 | 18 | 85 |
| 98 | 07/12/2022 | 15 | 5 |
| 99 | 08/12/2022 | 21 | 70 |
| 100 | 08/12/2022 | 19 | 10 |
| 101 | 09/12/2022 | 12 | 175 |
| 102 | 12/12/2022 | 2 | 75 |
| 103 | 13/12/2022 | 18 | 60 |
| 104 | 13/12/2022 | 14 | 110 |
| 105 | 13/12/2022 | 15 | 45 |
| 106 | 13/12/2022 | 9 | 5 |
| 107 | 14/12/2022 | 13 | 65 |
| 108 | 15/12/2022 | 5 | 65 |
| 109 | 15/12/2022 | 8 | 30 |
| 110 | 15/12/2022 | 1 | 125 |
| 111 | 19/12/2022 | 3 | 35 |
| 112 | 19/12/2022 | 20 | 70 |
| 113 | 19/12/2022 | 19 | 20 |
| 114 | 19/12/2022 | 10 | 2 |
| 115 | 19/12/2022 | 6 | 45 |
| 116 | 20/12/2022 | 17 | 20 |
| 117 | 20/12/2022 | 12 | 50 |
| 118 | 20/12/2022 | 18 | 5 |
| 119 | 20/12/2022 | 4 | 10 |
| 120 | 20/12/2022 | 9 | 5 |
| 121 | 10/1/2023 | 12 | 65 |
| 122 | 11/1/2022 | 20 | 125 |
| 123 | 11/1/2022 | 16 | 25 |
| 124 | 12/1/2022 | 11 | 10 |
| 125 | 12/1/2022 | 5 | 5 |
| 126 | 12/1/2022 | 16 | 135 |
| 127 | 13/1/2022 | 13 | 100 |
| 128 | 13/1/2022 | 2 | 5 |
| 129 | 13/1/2022 | 18 | 5 |
| 130 | 14/1/2022 | 12 | 100 |
| 131 | 16/1/2022 | 8 | 75 |
| 132 | 17/1/2022 | 18 | 50 |
| 133 | 17/1/2022 | 15 | 60 |
| 134 | 17/1/2022 | 14 | 5 |
| 135 | 17/1/2022 | 9 | 5 |
| 136 | 17/1/2022 | 17 | 45 |
| 137 | 18/1/2022 | 6 | 120 |
| 138 | 18/1/2022 | 21 | 80 |
| 139 | 19/1/2022 | 12 | 70 |
| 140 | 19/1/2022 | 19 | 55 |
| 141 | 19/1/2022 | 10 | 60 |
| 142 | 21/1/2022 | 12 | 150 |
| 143 | 23/1/2022 | 11 | 20 |
| 144 | 23/1/2022 | 5 | 100 |
| 145 | 24/1/2022 | 13 | 130 |
| 146 | 24/1/2022 | 3 | 130 |
| 147 | 25/1/2022 | 18 | 305 |
| 148 | 26/1/2022 | 12 | 90 |
| 149 | 26/1/2022 | 7 | 25 |
| 150 | 27/1/2022 | 5 | 75 |
| 151 | 27/1/2022 | 12 | 70 |
| 152 | 31/1/2022 | 10 | 110 |
| 153 | 31/1/2022 | 20 | 35 |
| 154 | 01/2/2022 | 12 | 45 |
| 155 | 02/2/2022 | 21 | 5 |
| 156 | 02/2/2022 | 19 | 35 |
| 157 | 03/2/2022 | 10 | 165 |
| 158 | 04/2/2022 | 13 | 185 |
| 159 | 05/2/2022 | 10 | 170 |
| 160 | 06/2/2022 | 10 | 30 |
| 161 | 07/2/2022 | 12 | 70 |
| 162 | 07/2/2022 | 18 | 45 |
| 163 | 07/2/2022 | 2 | 30 |
| 164 | 08/2/2022 | 13 | 130 |
| 165 | 15/2/2022 | 12 | 170 |
| 166 | 17/2/2022 | 10 | 40 |
| 167 | 22/2/2022 | 12 | 305 |
| 168 | 23/2/2022 | 10 | 535 |
| 169 | 24/2/2022 | 12 | 130 |
| 170 | 25/2/2022 | 12 | 315 |
| 171 | 26/2/2022 | 12 | 110 |
| 172 | 27/2/2022 | 12 | 5 |
| 173 | 28/2/2022 | 12 | 115 |
| 174 | 01/3/2022 | 12 | 90 |
| 175 | 02/3/2022 | 19 | 35 |
| 176 | 02/3/2022 | 5 | 50 |
| 177 | 03/3/2022 | 2 | 15 |
| 178 | 03/3/2022 | 12 | 110 |
| 179 | 06/3/2022 | 8 | 70 |
| 180 | 07/3/2022 | 13 | 30 |
| 181 | 08/3/2022 | 5 | 140 |
| 182 | 08/3/2022 | 19 | 55 |
| 183 | 08/3/2022 | 12 | 90 |
| 184 | 09/3/2022 | 21 | 5 |
| 185 | 09/3/2022 | 16 | 45 |
| 186 | 09/3/2022 | 20 | 65 |
| 187 | 10/3/2022 | 12 | 95 |
| 188 | 14/3/2022 | 12 | 65 |
| 189 | 14/3/2022 | 15 | 60 |
| 190 | 14/3/2022 | 9 | 35 |
| 191 | 14/3/2022 | 2 | 5 |
| 192 | 14/3/2022 | 14 | 15 |
| 193 | 14/3/2022 | 4 | 45 |
| 194 | 15/3/2022 | 12 | 15 |
| 195 | 15/3/2022 | 15 | 55 |
| 196 | 15/3/2022 | 17 | 50 |
| 197 | 15/3/2022 | 10 | 45 |
| 198 | 16/0/2022 | 21 | 75 |
| 199 | 16/3/2022 | 10 | 80 |
| 200 | 16/3/2022 | 12 | 45 |
| 201 | 17/3/2022 | 18 | 320 |
| 202 | 23/3/2022 | 10 | 390 |
| 203 | 23/3/2022 | 12 | 390 |
| 204 | 24/3/2022 | 12 | 65 |
| 205 | 24/3/2022 | 16 | 10 |
| 206 | 24/3/2022 | 18 | 45 |
| 207 | 24/3/2022 | 19 | 40 |
| 208 | 24/3/2022 | 20 | 15 |
| 209 | 24/3/2022 | 21 | 5 |
| 210 | 27/3/2022 | 21 | 30 |

**Table 3: Characteristics of the interview dataset**

| **Date** | **Ref** | **CR pseudonym** | **Duration (minutes)** | **Interviewee** | **Interview focus** |
| --- | --- | --- | --- | --- | --- |
| 2/9/2022 | 19 | Katekani Mabaso | 29 | Primary caregiver (Fatima Chauke) | Daily experience as paid caregiver, experiences of abuse. |
| 5/9/2022 | 20 | Ponisa Ngubeni | 33 | Primary caregiver (Enelo Ngubeni) | Caring for grandmother during hallucinations |
| 19/10/2022 | 20 | Ponisa Ngubeni | 9 | Primary caregiver (Enelo Ngubeni) | Improvements in grandfather’s health conditions |
| 2/11/2022 | 19 | Katekani Mabaso | 29 | Primary caregiver (Fatima Chauke) | Daily experience as paid caregiver, experiences of abuse. |
| 3/11/2022 | 10 | Samson Nkuna | 23 | Primary caregiver (Doris Nkuna) | Experience accessing eye health services for self at the district hospital. |
| 11/11/2022 | 13 | Adam Ubisi | 28 | Primary caregiver (Dorothy Mkhantswa) | Experience accessing radiological services for care recipient at the district hospital. |
| 15/11/2022 | 4 | WelaniSilaule | 26 | Primary caregiver (Isaac Silaule) | Faith-based caregiving practices. |
| 9/12/2022 | 12 | Xiluva Shabangu | 34 | Primary caregiver (VangamaNdubane) | Daily caregiving experiences, improvements to care recipient’s health status. |
| 13/12/2022 | 14 | Reason Mnisi | 34 | Primary caregiver (Hletisani Dlamini) | Negotiating time away from caregiving. |
| 14/1/2023 | 12 | Xiluva Shabangu | 50 | Two non-primary co-resident caregivers, Xiluva’s grandnephew (Mpho) and great-grandnephew (Sandziso) | Familial caregiving arrangements and family conflict. |
| 18/1/2023 | 6 | Rose Mhlanga | 67 | Primary caregiver (LulekaManyike) | Care recipient memory problem, motivations for caregiving. |
| 21/1/2023 | 12 | Xiluva Shabangu | 62 | Non-primary non-resident caregiver, Xiluva’s lastborn daughter (Kayise Shabangu) | Familial caregiving arrangements, family conflict, reasons for withdrawing from primary caregiver role. |
| 24/1/2023 | 3 | Hlekani Mnisi | 24 | Primary caregiver (Masana Mnisi) and care recipient (Hlekani Mnisi) | Caregiving when Hlekani experienced diabetic coma at night |
| 25/1/2023 | 18 | Rhandzu Mkhantswa | 44 | Primary caregiver (Xisthembiso Nkuna) | Care recipient’s memory problems, caregiving during episodes of disorientation and confusion. |
| 4/2/2023 | 13 | Adam Ubisi | 52 | Non-primary, co-resident caregiver and Adam’s grandniece (Minenhle) | Caregiving experiences, living away while Adam seeks therapy. |
| 4/2/2023 | 13 | Adam Ubisi | 14 | Non-primary, non-resident caregiver and Adam’s grandniece (Phetho) | Caregiving experiences. |
| 8/2/2023 | 13 | Adam Ubisi | 102 | Non-primary, non-resident caregiver and Adam’s niece (Dolly) | Caregiving experiences, family conflict. |
